# Functional characterization of T-cells from palatine tonsils in patients with chronic tonsillitis

**DOI:** 10.1371/journal.pone.0183214

**Published:** 2017-09-06

**Authors:** Katharina Geißler, Robby Markwart, Robert Pascal Requardt, Cynthia Weigel, Katja Schubert, André Scherag, Ignacio Rubio, Orlando Guntinas-Lichius

**Affiliations:** 1 Department of Otorhinolaryngology, Jena University Hospital, Jena, Germany; 2 Integrated Research and Treatment Center, Center for Sepsis Control and Care, Jena University Hospital, Jena, Germany; 3 Research Group Clinical Epidemiology, Integrated Research and Treatment Center, Center for Sepsis Control and Care, Jena University Hospital, Jena, Germany; 4 Institute for Molecular Cell Biology, Center for Molecular Biomedicine, Jena University Hospital, Jena, Germany; University of Iowa, UNITED STATES

## Abstract

The palatine tonsils, localized in the oropharynx, are easily accessible secondary lymphoid tissue in humans. Inflammation of the palatine tonsils, local and chronic in case of chronic tonsillitis (CT) or acute in the presence of a peritonsillar abscess (PTA), ranks among the most common diseases in otolaryngology. However, the functionality of tonsillar immune cells, notably T-cells, in the context of these immune pathologies is poorly understood. We have examined the functional status of human tonsillar T-cells in CT and compared it to the acute inflammatory setting of a PTA. Patients presenting with CT (n = 10) or unilateral PTA (n = 7) underwent bilateral tonsillectomy and a subgroup of 8 patients underwent additional blood sampling. T-cells were purified via automated magnetic selection and subjected to flow cytometry-based immunophenotyping. In addition, the response to T-cell receptor (TCR) stimulation was assessed at the level of proximal signaling, activation marker expression and proliferation. We observed no difference between the percentage of T helper (CD4(+)) cells from tonsil tissue in CT and PTA, but observed a trend towards a higher percentage of T helper cells in the blood of patients with PTA versus CT, probably reflecting an acute, systemic bacterial infection in the former cohort. Tonsils from CT harbored more PD-1(+) CD4(+) T-cells, pointing to T-cell exhaustion due to chronic infection. This notion was supported by functional studies that showed a tendency to weaker TCR responses of tonsillar T-cells from CT. Intriguingly, tonsillar T-cells recurrently featured a dampened response to T-cell receptor stimulation at the level of receptor proximal signaling steps compared to peripheral T-cells. In sum, our study documents distinct differences in tonsillar T-cell class distribution and function between the various pathological conditions. Our observations are consistent with the concept that tonsillar T-cells react to infections by eliciting specific immunological responses in chronic versus acute settings of inflammation.

## Introduction

### Palatine tonsils and inflammatory diseases

The palatine tonsils are located at the entrance of the upper aerodigestive tract for immune protection against ingested and inhaled pathogens. Immune protection in this area depends on both innate nonspecific defense mechanism and adaptive specific immune reactions. T-cells, in particular, are present in high numbers in palatine tonsils and are largely located in the extra-follicular spaces [1]. Given their lymphoid nature and as supported by a number of immunological studies it has been proposed that tonsils are inductive sites for humoral and cell-mediated immune responses [2]. For example, Tonsils have recently been described as sites of induction of oral immune tolerance [3]. However, there is an unsettled debate as to whether human tonsils contribute significantly to infection control or rather represent obsolete and futile immune entities. Beyond its conceptual importance, this issue is of high clinical relevance in the light of the high numbers of tonsillectomy surgeries performed as the result of various types of infectious complications.

Chronic tonsillitis (CT) is a common chronic inflammation of the palatine tonsils often requiring surgical excision of the affected tissue [4]. Criteria for tonsillectomy are at least 3 episodes of tonsillitis per year [5], which often conditions the need of antibiotic treatment. Patients with CT report about pain in throat and head, fatigue, fever, *foetor ex ore*, and cervical lymphadenopathy. Potential and partially serious complications include sepsis, rheumatic fever, endocarditis, glomerulonephritis and retropharyngeal or peritonsillar abscesses.

Peritonsillar abscesses (PTA) can occur as a complication of an acute severe tonsillitis. PTA describes a localized deep neck infection that develops in the peritonsillar space [6]. The infection can progress to airway obstruction, abscess rupture and asphyxia by aspiration of pus and necrosis resulting in sepsis or hemorrhage.

In a third form of tonsillar complication denominated tonsil hyperplasia (HY), the lymphoid tissue proliferates and grows in the absence of an ostensible episode of infection or inflammation for reasons that remain largely obscure. The typical age for this process is between 2 and 6 years. Notably, this process is irreversible in the sense that once HY has occurred, tonsils will not shrink to regain their original volume. Although HY itself is not a disease, obstructive HY can eventually cause afflictions such as snoring and sleep apnea.

CT and PTA or some therapy-refractory systemic diseases, for example certain forms of psoriasis or IgA-nephropathy [7, 8], are common indications for tonsillectomy. Indeed, tonsillectomy is one of the most common surgical procedures in young adults and infants (e.g. 737 000 tonsillectomies in the United States in 2006 [9]; in Germany 84.332 tonsillectomies in 2013 [10]). Although the discussions on the role and importance of tonsils for the immune response are controversial, a significant contribution of the tonsil palatina to immunity is supported by a number of studies, which described an association between tonsillectomy and increased susceptibility to infections. For example, in a nationwide Taiwanese cohort study Wang et al. documented a higher risk for deep throat infection after tonsillectomy [11]. Along these lines, a nationwide Danish cohort study detected a higher risk of Hodgkin lymphoma for tonsillectomized patients and an association to the mere diagnosis of tonsillitis [12]. Moreover, a Swedish study hypothesized that the incidence of a group of autoimmune diseases was higher in tonsillectomized individuals. A possible explanation for this association could be an immune dysfunction due to tonsillectomy [13]. Interestingly, tonsils have been proposed to be the sites responsible for the induction of peripheral tolerance to oral antigens [3], suggesting that tonsils may indeed be relevant for curbing immune function in particular infection scenarios.

In contrast to the previous reports a number of studies and meta-analysis did not observe significant immune alterations following tonsillectomy. Thus, a systemic review and meta-analysis of 35 studies including 1997 patients concluded that tonsillectomy has no clinically relevant negative effect on the immune system [14]. Furthermore, Nasrin et al. found that after tonsillectomy the humoral immune system was not significantly altered [15]. Hu et al. concluded that the long-term immune function did not decline after tonsil and adenoid resection in children [16].

Despite all these appreciations, there is an ongoing debate as to the net benefit of tonsils as a functional lymphoid organ and their contribution to infection control in humans. This controversy is not least fostered by the paucity of solid experimental data on the immunological status of tonsillar immune cells in the context of the various inflammatory conditions that can afflict the tonsil. Indeed, while a number of descriptive studies illustrate that human tonsils are equipped with all cellular components needed to elicit an immunological response to antigen (i.e. antigen presenting cells, T-cells, accessory cells, etc.) [2], there is very limited insight on how tonsillar immune cells respond functionally to any given inflammatory or infectious insult. We have conducted the present study to better understand the functional status of human tonsillar T-cells in human CT. In addition to CT we performed parallel sampling from PTA (as an example for an acute severe infection scenario) and from patient blood in order to understand or identify functional features associated to a chronic (tonsillitis) versus acute (abscess) infectious condition. Our findings evidence differences in the functional pattern of T-cells from these distinct settings that will be discussed in the context of the general debate regarding the immune functionality of human tonsils.

## Materials and methods

### Patients and tonsillectomy

Tonsillectomy was performed in the department of otorhinolaryngology of the Jena University Hospital, Germany. The patients were recruited between September 2013 and September 2014. The study protocol conformed to the ethical guidelines of the 1975 Declaration of Helsinki and was approved by the ethical review committee from the medical faculty of the Friedrich-Schiller-University Jena (No. 3972-01/14). We examined 12 male and 8 female patients aged 18 to 54 years (median 31 y) with CT, PTA or tonsil hyperplasia (HY). Specifically, patient numbers were as follows: 10 patients with CT (thereof 3 male, median age: 24.7 y), 7 patients with PTA (thereof 6 male, median age: 38.6 y) and 3 patients with HY (all 3 male, median age: 34.7 y). All 3 patients with HY underwent tonsillectomy due to obstructive sleep apnea syndrome. Despite having been able to recruit only n = 3 patients with HY we include the data from this group since HY represents the best approximation to a healthy, infection-free control tonsil group. For CT we included patients with 3 or more episodes of acute tonsillitis within a year that had been treated with antibiotics. All cases of CT were diagnosed on the basis of a proven bacterial infection. All patients underwent bilateral tonsillectomy, i.e. in patients with PTA both the side with the abscess and the healthy contralateral side were removed. Blood was additionally drawn (5 x 9ml EDTA tubes, taken during surgery) from some of the patients (2 patients with CT, all 7 patients with PTA, 2 patients with HY). Exclusion criteria for all groups were steroid therapy and other immunosuppressive therapy, severe chronic diseases in the medical history and therapy with anticoagulants or coagulation values lying under the norm. The surgery of patients with CT and HY was carried out in an inflammation-free interval, whereas tonsillectomy in patients with PTA was performed during a phase of acute inflammation with increased systemic inflammatory markers (e. g. increased C-reactive protein). Tonsillectomy was always performed in the morning between 10.00 am and 12.00 am.

Owing to the high number of highly material-consuming functional assays performed with the purified tonsillar T-cells, not all tonsil samples did provide sufficient lymphocytes to perform all assays.

### Materials, reagents and peptides

Dead Cell Stain Kit and 70 μM cell strainer were from BD Pharmingen (Franklin Lakes, USA); Sytox AADvancedTM from Life Technologies Corporation (Carlsbad, USA); RPMI 1640 medium and PBS were purchased from Biochrome AG (Berlin, Germany); Albumin fraction V, β-mercaptoethanol, Acrylamide-bisacrylamide stock solution 37,5:1, magnesiumchloride, methanol, N-(2-Hydroxyethyl)piperazine-N'-(2-ethane-sulfonic-acid) were from Carl Roth GmbH & Co. KG (Karlsruhe, Germany); Streptavidin from Dianova (Hamburg, Germany); Phorbol-12-myristate-13-acetate and Microcystine from Enzo Lifescience Inc. (Lörrach, Germany); Leucosep Falcons from Greiner Bio One International AG (Frickenhausen, Germany); Cell culture plates were from Corning Incorporated (Corning, USA); Click-iT EdU Alexa Fluor 488 Flow Cytometry Assay Kit from Life Technologies Corporation (Carlsbad, USA); CD4 and CD8 MicroBeads, Inside Stain Kit, T-Cell Activation/Expansion Kit and Treg Detection Kit (CD4/CD25/FoxP3) were all from Miltenyi Biotec (Bergisch Gladbach, Germany); S-Monovetten (9 ml K3E) were from Sarstedt (Nürnbrecht, Germany); Brefeldin A, Ionomycine and Histopaque-1077 from Sigma-Aldrich & Co Inc. (St. Louis, USA); N-Dodecyl-β-D-Maltoside was purchased from EMD Chemicals Inc. (Gibbstown, USA); ProSieve 50 Gradient-Gel Solution from Lonza Group AG (Basel, Switzerland); FCS was purchased from Biowest LLC (Kansas City, USA); CFSE was acquired from Enzo Lifescience Inc. (Lörrach, Germany).

### Antibodies and flow cytometry

Antibodies for T-cell stimulation: anti-human CD3 Biotin (clone OKT3), anti-human CD28 Biotin (clone CD28.2), anti-human CD28 purified (clone CD28.2) and anti-human CD3 purified (clone OKT3) from eBioscience Inc. (San Diego, USA).

Antibodies for flow cytometry analysis: anti-human CD3-FITC (clone MEM-57), anti-human CD4-PE (clone MEM-241), anti-human CD8-APC (clone MEM-31), anti-human CD45RA-FITC (clone HI100) and anti-human CD62L-APC (clone LT-TD180) from ImmunoTools (Friesoythe, Germany); Anti-human CD152 (CTLA-4)-PE (clone 14D3) and anti-human CD279 (PD-1)-FITC (clone MIH4) from eBioscience Inc. (San Diego, USA); CD25-APC human (clone 4E3), CD69-APC human (clone FN50), CD154-APC human (clone 5C8), anti-FoxP3-PE human (clone 3G3) and anti-IL-2-PE human (clone N7.48A) were purchased from Miltenyi Biotec GmbH (Bergisch Gladbach, Germany).

Antibodies for Western blot analysis: Akt (pan) Rabbit mAb #4685, LAT antibody #9166, p44/42 MAPK (Erk1/2) Rabbit mAb #4695, Phospho-Akt (Ser473) (D9E) XP Rabbit mAb #4060, Phospho-p44/42 MAPK (Erk1/2) (Thr202/Tyr204) (D13.14.4E) XP Rabbit mAb #4370, Phospho- LAT (Tyr191) antibody #3584, Phospho-PLCγ1 (Tyr783) antibody #2821 and Phospho-Zap70 (Tyr319)/Syk (Tyr352) antibody #2701 from Cell Signaling Technology (Cambridge, Great Britain). PLCγ1 (1249) sc-81 and Zap70 (1E72) sc-32760 from Santa Cruz Biotechnology Inc. (Dallas, USA).

All flow cytometry data were acquired using a FACS Canto or FACS Calibur (BD PharmingenTM, Franklin Lakes, USA) and analyzed with FlowJo software (TreeStar Inc., Ashland, USA).

### T-cell preparation, isolation and purification

We have previously developed a protocol for T-cell purification from human tonsils including automated magnetic sorting [17]. Briefly, tonsils were processed no later than 2 hours upon tonsillectomy, a time lag during which they were kept in PBS on ice. Tonsil tissue was minced with scissors and homogenized by carefully squashing it through 70 μm cell strainers. After several rounds of washing the cell homogenate was resuspended in PBS/0.5% BSA/2 mM EDTA and equilibrated on a Ficoll gradient (Histopaque-1077; Sigma-Aldrich, St. Louis, USA) by centrifugation. The peripheral blood mononuclear cell (PBMC) layer was collected and diluted with PBS/0.5% BSA/2 mM EDTA. CD4(+) and CD8(+) T-cells were isolated from these fraction by automated magnetic positive selection using CD4 and CD8 Microbeads on the autoMACS Pro Separator device (Milteny Biotech, Bergisch Gladbach, Germany).

T-cell isolation from 45 ml EDTA-anticoagulated blood samples diluted in PBS followed the same major steps as described above.

### T-cell cultivation and TCR stimulation of human T-cells

T-cells were routinely cultured at a density of no less than 0.5x10^6^ cells/ml in RPMI 1640 medium supplemented with antibiotics and 10% heat-inactivated fetal calf serum (FCS). T-cells were transferred to medium supplemented with antibiotics and 3% heat-inactivated FCS and incubated 24 h prior to all presented T-cell functional assays, except proliferation and apoptosis measurements that were carried out in the presence of 10% FCS. Stimulation of the T-cell receptor (TCR) was accomplished using various protocols that differed in TCR activation strength and avidity. Stimulation in the absence of TCR clustering was performed using 1.65 μg/ml biotinylated anti-CD3 Ab and/or biotinylated anti-CD28 Ab each administered in solution. For high-affinity, high avidity stimulation the same Abs were additionally cross-linked in solution by supplementing 1.65 μg/ml streptavidine. Alternatively, T-cells were challenged with anti-CD2, anti-CD3 and anti-CD28 captured on anti-biotin MACSiBead particles (T-cell activation and expansion Kit, Miltenyi Biotec GmbH). Finally, T-cells were pharmacologically activated by combined addition of 75 μM phorbol ester (tetradecanoylphorbolacetate, TPA) and 500 μg/ml ionomycine.

### T-cell phenotyping via flow cytometric analysis

For flow cytometric characterization, 0.25x10^6^ T-cells cells per sample were washed twice with PBS/0.5% BSA/2 mM EDTA and stained with the appropriate Ab for 30 min on ice. Regulatory T-cells were detected using the Treg Detection Kit (Miltenyi Biotec GmbH) according to the manufacturer’s instructions. For intracellular staining of IL-2, T-cells were washed twice with PBS/0.5% BSA/2 mM EDTA, fixed and permeabilized using the Inside Stain Kit (Miltenyi Biotec GmbH) according to the manufacturer’s manual. Cells were stained with anti-IL-2 Ab at room temperature for 15 min. All antibodies were used in an appropriate concentration and fluorochrome combination. Samples were measured on a FACS Calibur (BD Pharmingen) and data were evaluated with FlowJo software (TreeStar Inc., Ashland, USA). In all applications, co-staining with Sytox AADvanced (Thermo Fisher Scientific, Waltham, USA) was performed in order to discriminate vital from dead cells.

### Proliferation assays

T-cells were labelled with 0.5 μM CFSE for 10 min at 37°C, washed with RPMI/10% FCS and placed on ice for 5 min. Cells were seeded at a density of 10^6^ cells/ml on 24-well plates. 6 days later T-cells were harvested, stained with Sytox AADvanced for live/dead cell discrimination and analyzed via flow cytometry.

DNA synthesis was analyzed 46 h after stimulation of the T-cells using the Click-iT EdU flow cytometry assay kit (Thermo Fisher Scientific, Waltham, USA) according to the manufacturer´s protocol.

### TCR signaling analysis

For analysis of TCR signal transduction T-cells were suspended in RPMI medium supplemented with 25 mM HEPES pH 7.5 and 1% BSA at a density of 3 x 10^6^ cells/ml and placed to rest on ice for one hour following isolation. T-cell suspensions (1 ml/assay point) were then moved to a 37°C water bath and stimulations were carried out as described above. To stop reactions cells were quickly spun down in a table-top centrifuge, the supernatant was removed and cell pellets were lysed in 250 μl ice-cold lysis solution (50 mM HEPES pH 7.5, 100 mM NaCl, 5 mM MgCl_2_, 1 mM EGTA, 1% NP-40, 0,1% *n*-dodecyl-β-D-maltoside, protease and phosphatase inhibitors). Lysates were vortexed, incubated 10 min at room temperature and 10 min on ice. Lysates were cleared by centrifugation and processed for gradient-gel electrophoresis and Western Blot analysis.

### Statistical analysis

We summarized all continuous read-outs of the immunophenotyping and functional assays by box-plots or bar charts. We applied exact non-parametric methods to address the small sample size and potential non-normality of the data. The following six comparisons were performed:

1. blood (blo) samples of patients with peritonsillar abscess (PTA-blo) vs. blood samples of patients with chronic tonsillitis (CT-blo);
2. PTA-blo vs. blood samples of patients with tonsil hyperplasia (HY-blo);
3. tonsil samples of patients with peritonsillar abscess one afflicted by the abscess (PTA-abs.ton) vs. tonsil sample of patients with chronic tonsillitis (CT-ton);
4. PTA-abs.ton vs. tonsil sample of patients with tonsil hyperplasia (HY-ton) and
5. the paired comparison of tonsil samples of patients with peritonsillar abscess one afflicted by the abscess and (PTA-abs.ton) vs. tonsil sample of the “healthy” counterpart (PTA-hea.ton).
6. the paired comparison of tissue and blood samples in the same patients stratified by disease and experimental condition (see S1 Table).

For the comparisons of two independent groups (a)-(d) we applied the exact Mann Whitney test; for the paired comparisons (e), (f) we used the Wilcoxon signed-rank test. As this was an explorative analysis, we applied an uncorrected significance level of α = 0.05 to screen for differences in the distributions. The analysis were performed using either the FlowJo software (TreeStar Inc., Ashland, USA), SAS 9.4. or R 3.3.2.

## Results

### Immune receptor/marker phenotyping

**Fig 1:**
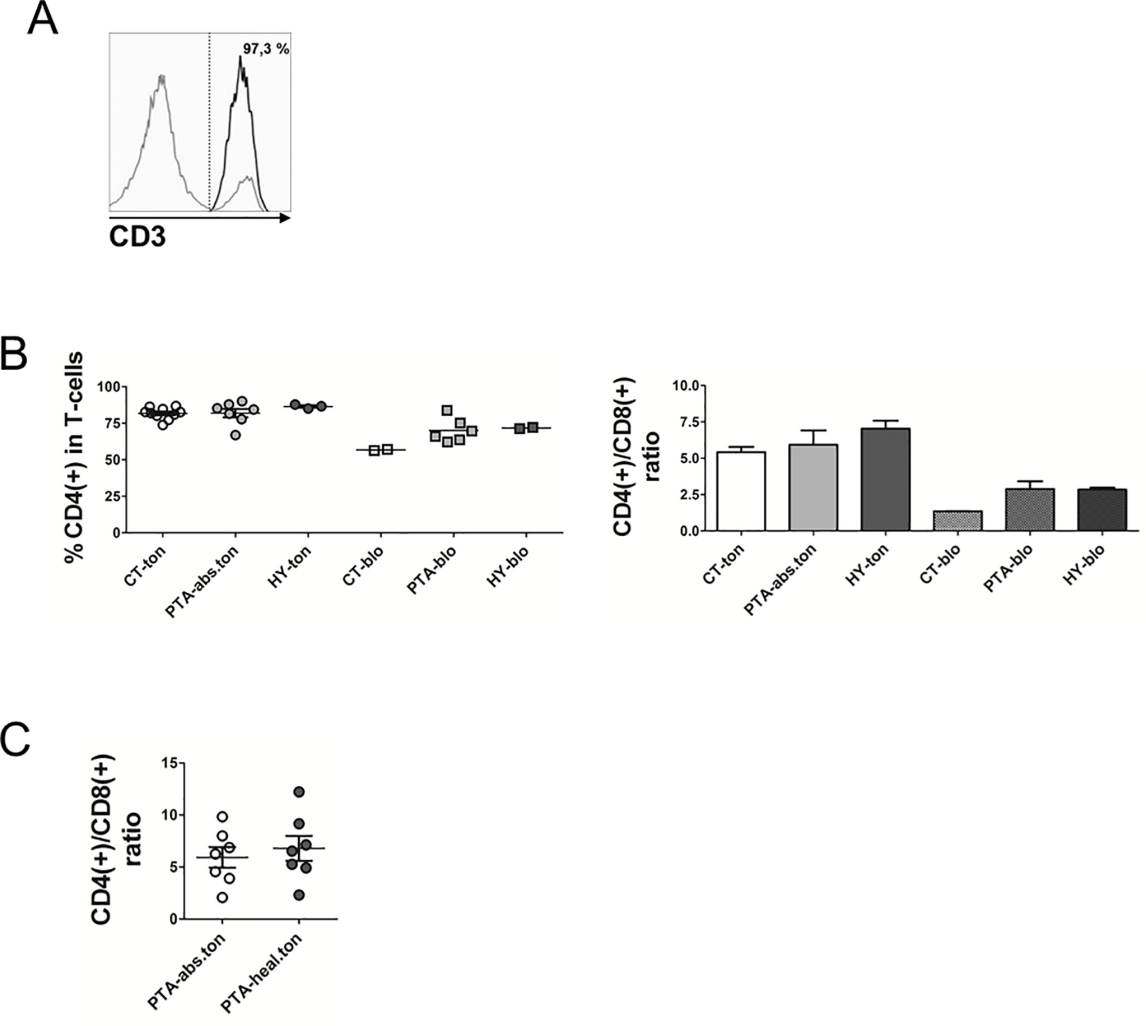
Tonsillar CD4/CD8 T-cell counts are not affected by the particular inflammation scenarios. **(A)** Purity of tonsillar T-cells isolated by positive magnetic selection as determined by surface CD3 staining. A representative flow cytometry histogram depicting CD3 staining of purified CD4(+)CD8(+) T-cells (black profile) and the residual negative cellular fraction (grey profile) is shown. Insert describes the purity in % CD3 positive of total tonsillar cells. **(B)** The surface expression of CD4 and CD8 on purified tonsillar and peripheral T-cells was assessed by flow cytometry and plotted as % CD4(+) of total T-cells and CD4(+)/CD8(+) ratio. For statistical analysis, see S1 Table. **(C)** CD4(+)/CD8(+) T-cell numbers from tonsils with abscess and their paired healthy counterparts. Data are plotted as CD4(+)/CD8(+) ratio. Data are presented as mean + SEM. CT = chronic tonsillitis; PTA = peritonsillar abscess; HY = tonsillar hyperplasia; ton = tonsil; blo = blood; abs = abscess; hea = healthy.

As a first step towards a functional characterization of human T-cells in palatine tonsils we determined the expression of immune phenotypic markers on CD4/CD8 T-cells. T-cells were isolated to high purity from palatine tonsils using a recently established purification routine involving sequential density gradient centrifugation followed by automated magnetic separation using the autoMACSPro system and anti-CD4/CD8 beads for positive selection [17]. The overall average yield was 15x10^6^ T-cells per gram tonsil tissue. The purity of T-cell preparations always exceeded 95% for tonsils from all backgrounds as scored by CD3 staining (Fig 1A). In line with previous studies [17], we detected a markedly higher proportion of tonsillar CD4(+) T-cells as compared to the average CD4/CD8 ratio of around 2 commonly found in the periphery or in spleen (Fig 1B). However, we observed no significant differences in the total numbers or in the CD4/CD8 ratios between tonsils of the various patient groups, suggesting that the various infection scenarios did not translate to large expansion or contraction of resident T-cell compartments. The higher proportion of CD4(+) helper T-cells in affected tonsils was not a consequence of the various infection/pathological scenarios because the paired, contralateral tonsils from PTA-patients harbored the same T-cell numbers (Fig 1C), as did tonsils from HY (Fig 1B). It is also worth to note that patients with PTA or HY featured reproducible increases in the numbers of CD4(+) helper T-cells in the periphery (Fig 1B), reminiscent of lymphocytosis described for cases of PTA associated to infectious mononucleosis [18]. In contrast, the blood from CT patients harbored average numbers and proportions of CD4(+) and CD8(+) lymphocytes. In sum, we concluded that CT did not result in altered CD4/CD8 T-cell ratios in the tonsils while the increased CD4 numbers in the blood from PTA patients likely reflected an acute infectious scenario.

### Tonsils from CT and PTA show no variations in their naïve/effector T-cell reservoir

**Fig 2:**
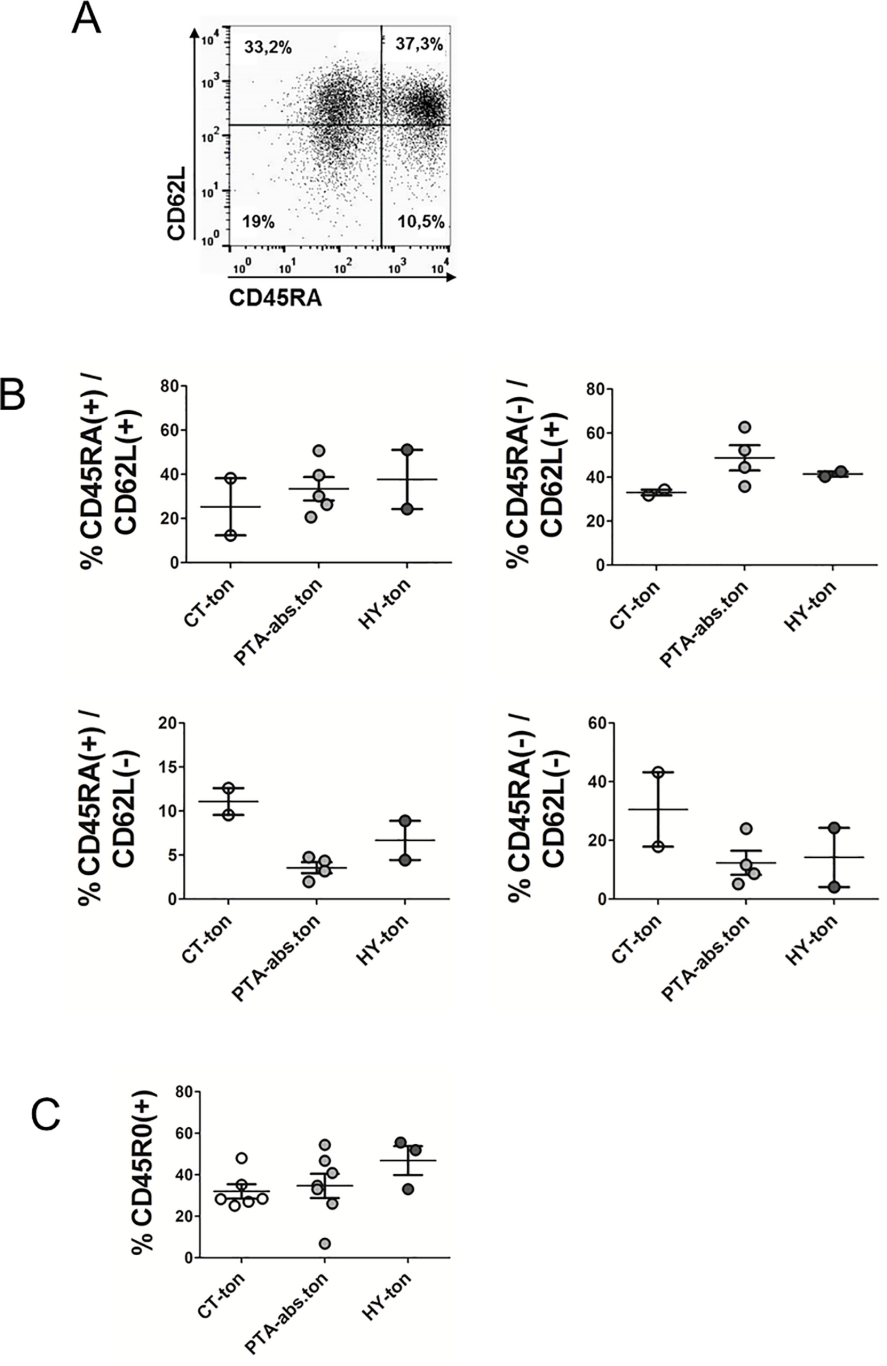
Tonsillar T-cell immune phenotype. CD4(+) plus CD8(+) T-cells were purified from tonsillar samples followed by fluorescence assisted immune staining of CD45RA and CD62L for the determination of naïve/effector/memory T-cells. All shown measurements were performed on the mixed CD4(+)CD8(+) preparations. See text for further details. **(A)** Representative histogram of a double staining for CD45RA and CD62L. **(B)** Quantification of all single and double positive CD45RA/CD62L populations for the various tonsillar disease groups. **(C)** Flow cytometric determination of CD45R0 expression on tonsillar T-cells. CT = chronic tonsillitis; PTA = peritonsillar abscess; HY = tonsillar hyperplasia; ton = tonsil; abs = abscess.

In order to dissect standard naïve/effector/memory T-cell populations in the tonsil we stained for CD45RA and CD62L. The common presence of both CD45RA and CD62L is routinely used to identify naïve T-cells. The absence of both markers is characteristic of effector memory T-cells while the singular expression of CD45RA or CD62L is associated to effector T-cells and central memory T-cells, respectively (Fig 2A) [19]. As seen in Fig 2B this analysis evidenced a trend to higher numbers of effector T-cells and effector memory T-cells in tonsils of CT patients, although these differences did not achieve statistical significance. This accumulation of effector T-cells may reflect a continuous exposure to antigen in the tonsils from CT patients [20–23]. To collect more evidence regarding the fate of T-cell memory in tonsils, we also stained for CD45R0, another established marker of memory T-cells (Fig 2C). We found no differences in the proportion of CD45R0 positive tonsillar T-cells among the various groups, corroborating that memory T-cell populations had not settled or exited the tonsil under any of the clinical manifestations. Finally, we also checked whether tonsils had elevated numbers of tissue–resident memory cells, a population characterized by upregulated levels of CD11a. These cells, which have been recently detected in numerous human tissues and epithelia, can react to secondary antigen encounters and are considered a first-line of defense against invading pathogens at mucosal barriers [24]. We detected no significant differences in the proportions of this T-cell population among the various groups (data not shown), confirming the previous findings.

### Features of immune suppression in tonsils: Assessing regulatory T-cell numbers and inhibitory cell surface receptors

**Fig 3:**
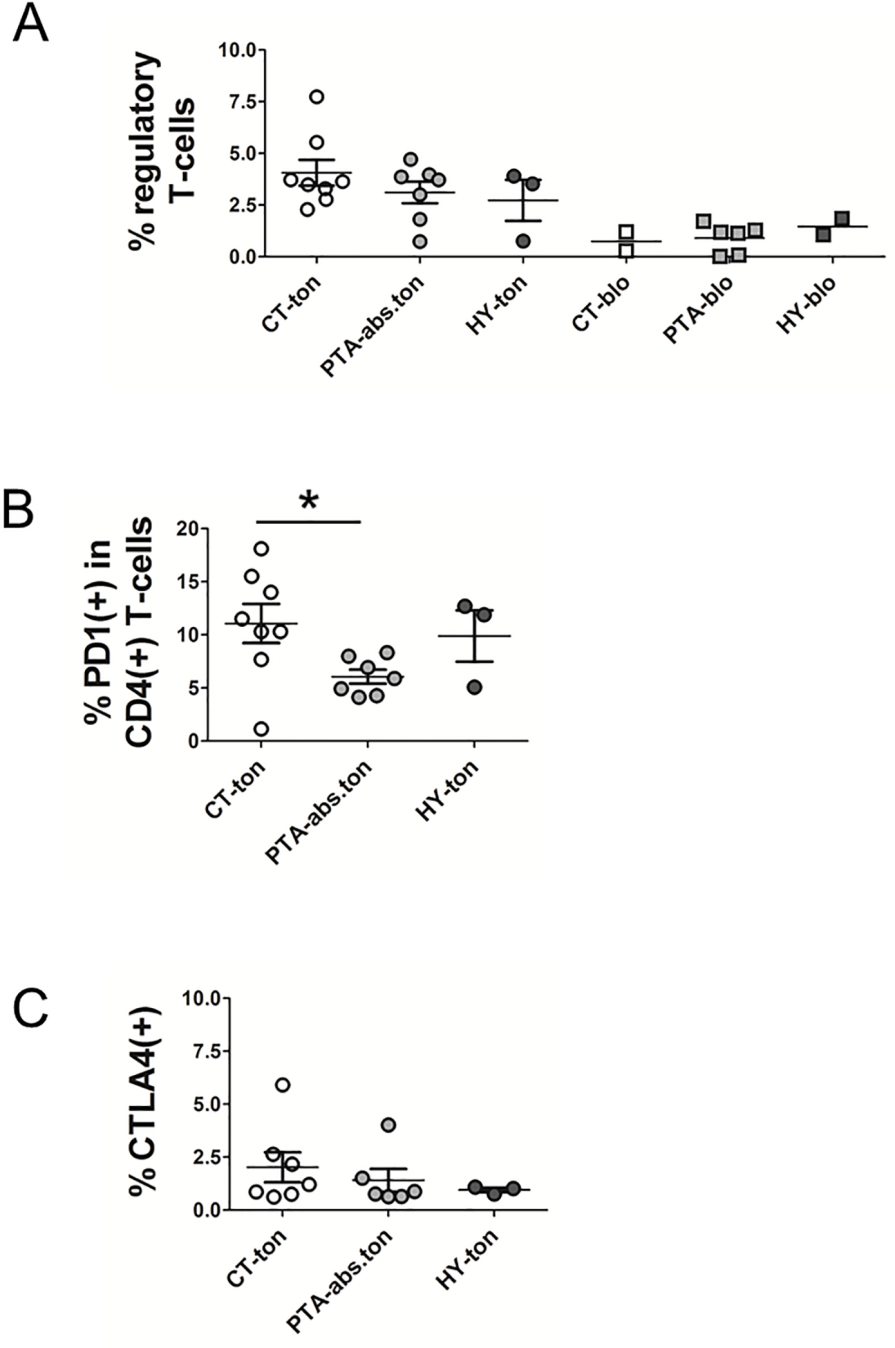
Inhibitory and suppressed T-cell populations in tonsils. CD4(+) and CD8(+) T-cells purified from tonsillar samples were subjected to fluorescence assisted immune phenotyping for the determination of inhibitory T-cell subgroups. All shown measurements were carried out on the mixed CD4(+)CD8(+) preparations. **(A)** The incidence of regulatory T-cells (Tregs) was determined by triple CD4/CD25/FoxP3 staining and plotted as percentage of Tregs in all T-cells both for tonsillar and blood samples. For statistical analysis, see suppl.S1 Table. **(B)** The fraction of PD-1 expressing T-cells was determined by flow cytometric determination of PD-1 cell surface staining and plotted as percentage of PD-1(+) cells in CD4(+) lymphocytes (CT-ton vs. PTA-abs.ton p = 0.026). **(C)** The surface expression of CTLA-4 on tonsillar T-cells was determined by flow cytometry and plotted as percentage of CTLA4(+) in all T-cells. * indicates comparisons that met an unadjusted p≤0.05 (explorative significance level). CT = chronic tonsillitis; PTA = peritonsillar abscess; HY = tonsillar hyperplasia; ton = tonsil; abs = abscess.

The higher proportion of effector T-cells in tonsils from CT was indicative of past episodes of antigen-dependent activation of T-cells in the tonsils. Exposure to antigen can initially cause the accumulation of antigen specific effector T-cells. However, a chronic infection can eventually lead to the exhaustion of pathogen-specific effector T-cells and a concomitant immune suppression or tolerance, as described for numerous infection settings [20–23]. The accumulation of inhibitory immune cell populations such as regulatory T-cells (Tregs) and the up-regulation of inhibitory receptors on the T-cell surface have been put forward as two mechanisms of immune suppression in the aftermath of hyper-inflammatory syndromes or in the course of chronic infections [25–27]. To understand whether tonsils from CT or tonsils in general featured any of these hallmarks of immune suppression we assessed Treg numbers in the tonsil and in paired blood samples by staining for CD4(+)CD25(+)FOXP3(+) in all T-cells (Fig 3A, statistical analysis is presented in S1 Table). In this analysis we observed that tonsils from all disease groups harbored higher fractional numbers of Tregs compared to blood. Moreover, within groups, a trend to more Tregs in tonsils from CT patients became evident. In sum, while the observed differences were minor only, we concluded that changes in Treg numbers were a potential cause for an immune suppressive environment, e.g. in the context of chronic tonsillitis.

In a second round of experiments we stained for the inhibitory receptors PD-1 and CTLA-4. Inhibitory immune receptors PD-1 and CTLA-4 are found to be up-regulated in multiple immune-suppressive conditions [28, 29]. We detected more numbers of T-cells with PD-1 expression in patients with CT (Fig 3B). T-cells from patients with CT, in average, expressed higher PD-1 levels (in PD-1(+)) on their surface than T-cells from peritonsillar abscess (PD-1(+) in CD4(+) T-cells p = 0.026). We also monitored the levels of a second major inhibitory receptor, CTLA-4, and found no significant differences in frequency or expression levels (Fig 3C). We conclude that patients with CT have an elevated count of PD-1(high) T-cells, a feature consistent with an environment of T-cell suppression.

### Naïve T-cells from patients with CT show signs of pre-activation

**Fig 4:**
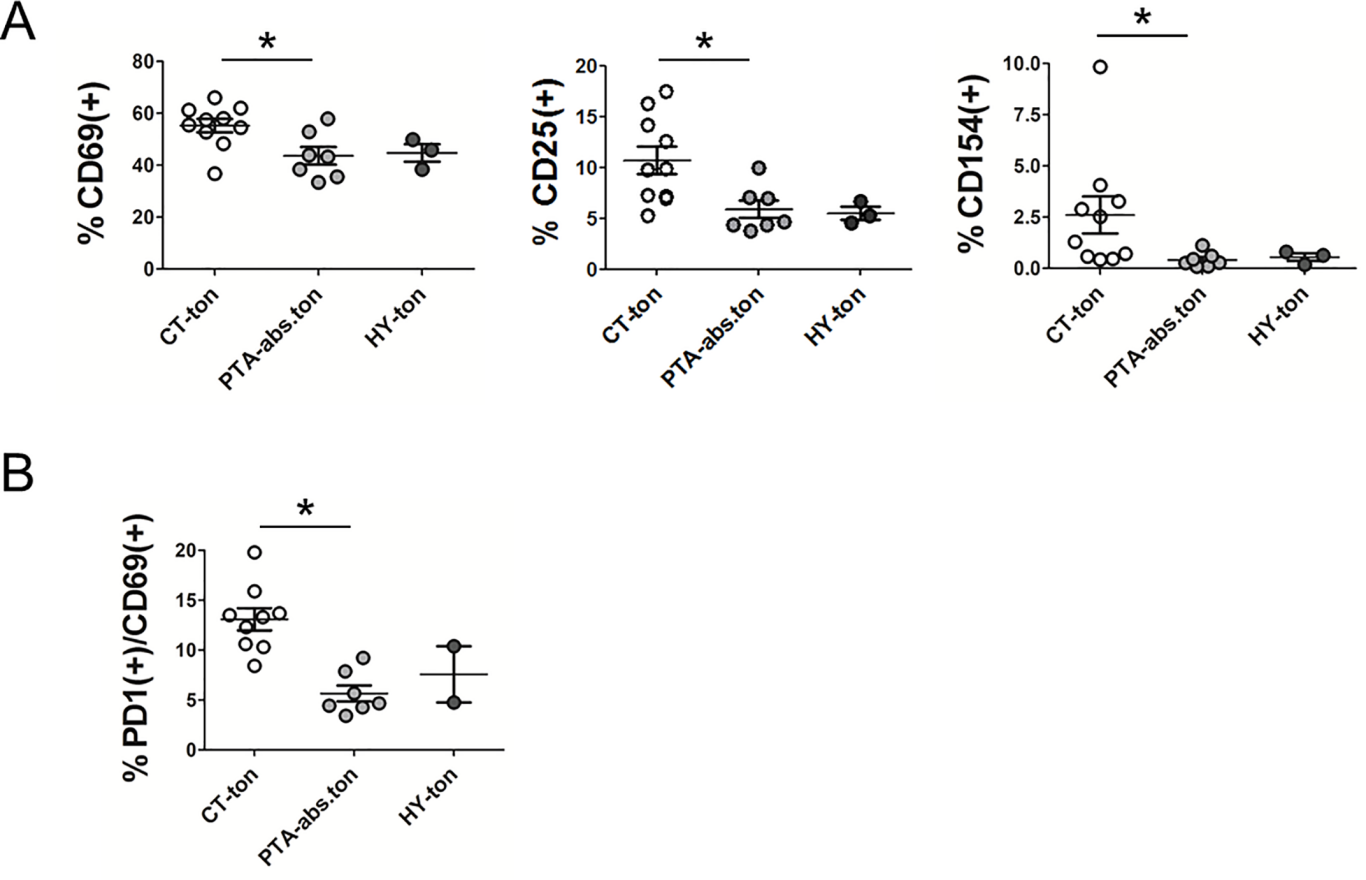
Tonsillar T-cells from chronic tonsillitis are basally activated. CD4(+)/CD8(+) T-cells were isolated from tonsils of the different disease scenarios and their activation status was determined via flow cytometry analysis of the mixed CD4(+)CD8(+) preparations. **(A)** CD69, CD25 and CD154 activation marker surface expression determined by flow cytometry and plotted as percentage of all T-cells. **(B)** Double immune-staining for PD-1 and CD69 in all T-cells. Only statistically significant groups are labelled (* p<0.05). CT = chronic tonsillitis; PTA = peritonsillar abscess; HY = tonsillar hyperplasia; ton = tonsil; abs = abscess.

The results presented above were consistent with an immune-suppressed status of T-cells in tonsils from CT. At the same time the higher numbers of effector T-cells and effector memory T-cells in tonsils from CT indicated that T-cells had experienced increased antigen exposure and activation within the tonsils. To address if T-cells had indeed a constitutively high basal level of activation in chronic tonsillitis, we compared the surface expression of CD69, CD25 and CD154 in freshly isolated tonsillar T-cells. All three proteins are involved in early steps of T-cell activation, become rapidly up-regulated and exposed on the T-cell surface following T-cell activation and are routinely monitored as T-cell activation markers. As shown in Fig 4A T-cells from tonsils of patients with CT featured higher expression of CD69, CD25 and CD154 compared to patients with peritonsillar abscess, consistent with a higher level of T-cell activation in CT. Of note, PD-1 overexpression was not overrepresented in the CD69(+) population (Fig 4B; compare with Fig 3B), suggesting that PD-1 up-regulation was not directly related to the current activation status of the T-cells.

### Tonsillar T-cells show an unaltered early activation response to TCR engagement

**Fig 5:**
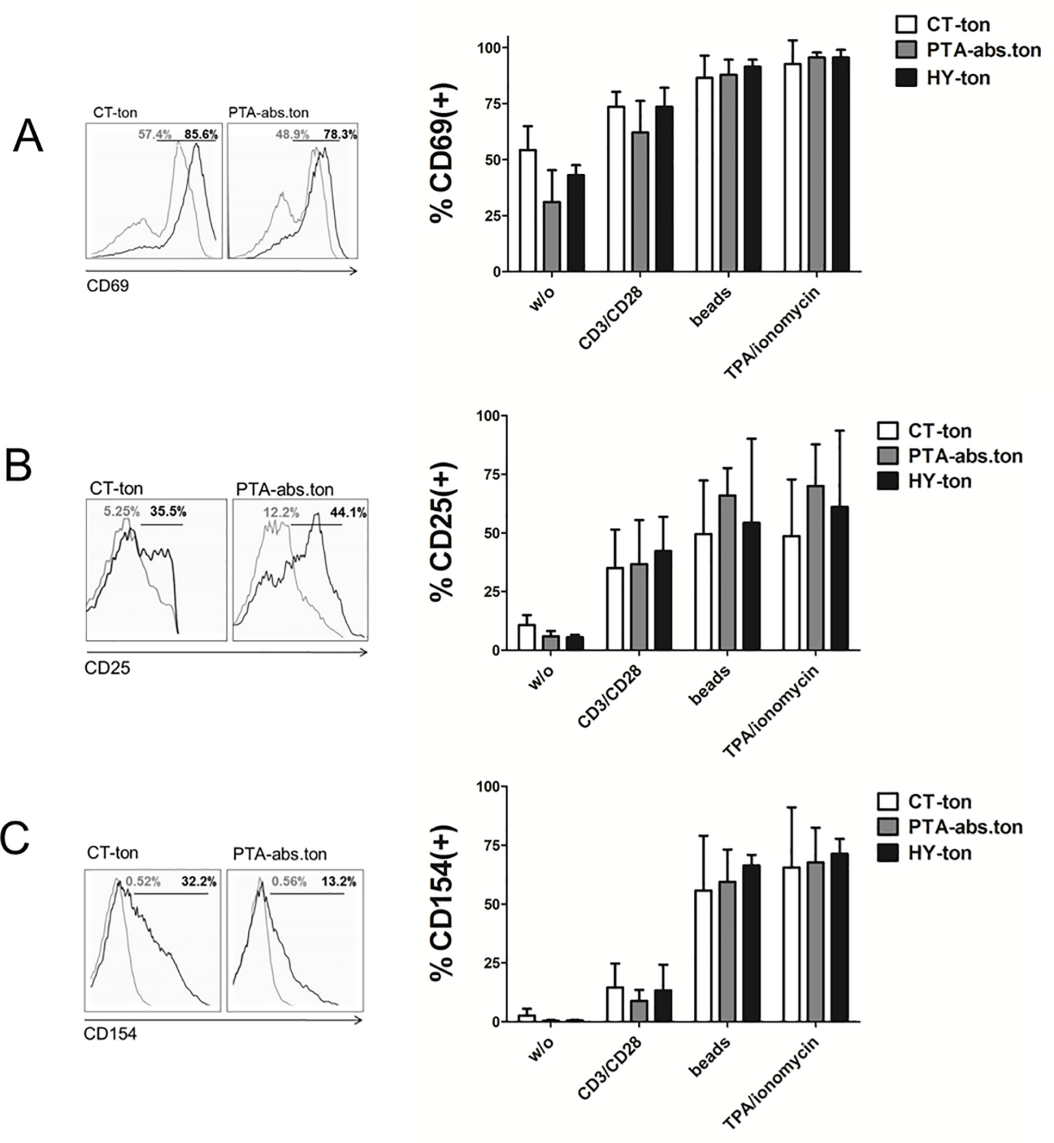
Tonsillar T-cells are not compromised in activation marker upregulation in response to TCR activation. CD4/CD8 T-cells isolated from tonsils were challenged with the indicated formulations of anti CD3 and/or CD28 Abs or with a mix of phorbol ester and Ionomycine (TPA/Iono) and subjected to flow cytometric determination of activation marker CD69 **(A)**, CD25 **(B)** and CD154 **(C)** expression, always plotted as percentage of all T-cells. All measurements were carried out on the mixed CD4(+)CD8(+) preparations. Representative fluorescence profiles for stimulated (black line) *versus* non-stimulated samples (grey curves) are shown on the left side of each panel. Beads: anti-CD3 and anti-CD28 Abs immobilized on beads. CT = chronic tonsillitis; PTA = peritonsillar abscess; HY = tonsillar hyperplasia; ton = tonsil; abs = abscess.

Tonsillar T-cells from CT featured a high basal activation state and signs of immune tolerance, i.e. the higher PD-1 score. To understand whether tonsillar T-cells could mount a functional response to an antigenic challenge we investigated the T-cell responses to TCR stimulation at the level of activation marker up-regulation (Fig 5). TCR stimulation was accomplished by applying activatory Abs for the CD3 epsilon chain of the TCR and the CD28 co-stimulatory receptor. Of note, anti-CD3 and anti-CD28 Abs were applied either in solution or immobilized on beads to provide additional clustering of TCR and CD28 molecules on the T-cells surface, a feature known to provide a more physiological T-cell activation [30, 31]. As shown in Fig 5A, 5B, and 5C T-cells from all three disease scenarios responded with a similar up-regulation of the three activation markers CD69, CD25 and CD154 to TCR stimulation. These findings showed that tonsillar T-cells from all points exhibited a comparable and undistinguishable early response to TCR stimulation.

### Receptor-proximal TCR signal transduction

**Fig 6:**
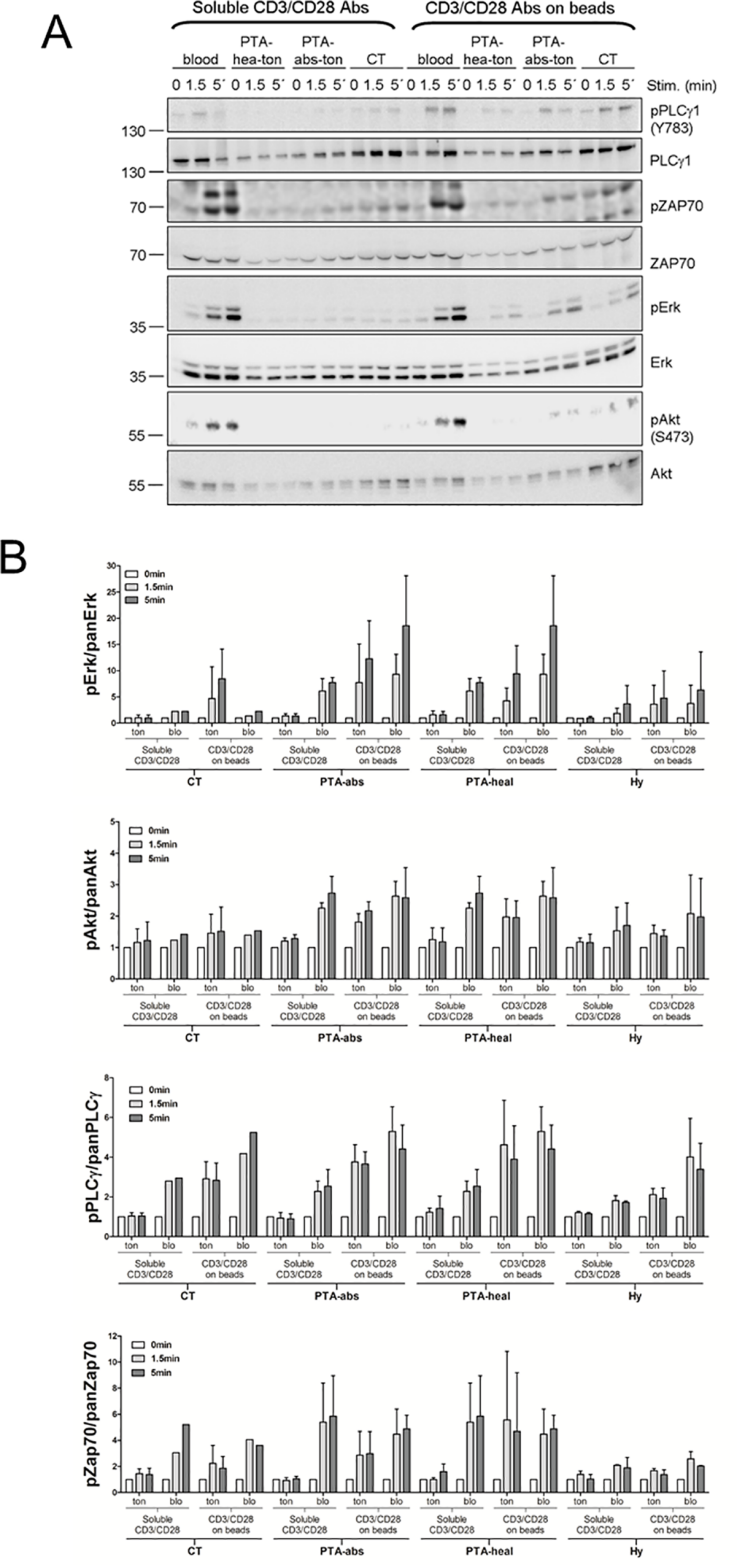
Analysis of TCR-proximal signal transduction in tonsillar T-cells. **(A)** CD4/CD8 T-cells were isolated from tonsils and resuspended in FCS-free medium. The total CD4(+)CD8(+) T-cell preparations were challenged with soluble or immobilized anti CD3/CD28 Abs to activate the TCR. Cells were lysed and cellular extracts were processed for Western Blot detection of total and phosphorylated versions of PLCγ, ZAP70, Erk and Akt. **(B)** Bands were quantified by densitometry and the ratio of phosphorylated to total protein was plotted as as fold activation of unstimulated samples (0 min). The quantification includes all measured tonsil samples. Note that the values for PTA-abs/blo and PTA-heal/blo represent perforce the same data and are plotted twice for illustrative reasons. Data are presented as mean + SEM. pPLCγ, pErk, pAkt and pZAP-70 mark the phosphorylated versions of the respective proteins. CT = chronic tonsillitis; PTA = peritonsillar abscess; HY = tonsillar hyperplasia; ton = tonsil; blo = blood; abs = abscess; hea = healthy.

Signals emanating from activated TCRs are propagated intracellularly by a complex network of signal transduction pathways. Physiological or pathological alterations in TCR signaling ultimately underlie changes or aberrancies in responsiveness and fate-decision taking of T-cells exposed to antigenic challenge. We took T-cells from patients with CT and PTA and compared the activation status of selected nodal signaling mediators following T-cell activation. These biochemical experiments required large amounts of T-cells (3x10^6^ T-cells per stimulation point) and could therefore only be performed in those cases, in which T-cell preparations from CT or PTA individuals resulted in an exceptionally high yield of T-cells (n = 5 for CT and n = 5 for PTA). T-cells were deprived of serum for 2 h to down-modulate global signaling and stimulated for 1.5 or 5 min with CD3/CD28 Abs applied in solution or immobilized on bead surfaces. Reactions were stopped by cell lysis on ice and cell extracts were processed for western blot analysis of 4 key mediators of the TCR signal: phospholipase-Cγ1 (PLCγ1), ZAP70, Erk and Akt. All 4 signaling intermediates are activated as a result of phosphorylation by upstream kinases and thus the activation status is determined using phosphorylation-site specific antibodies and building the ratio of phosphorylated to total protein. Fig 6A shows representative data obtained for tonsillar and peripheral T-cell preparations from the various clinical conditions and Fig 6B depicts the sum of all data sets. Over all, tonsillar T-cells showed a tendency to weaker responses to TCR stimulation than their counterparts from the periphery, irrespective of the clinical background, although these differences did not attain statistical significance (S1 Table). As a matter of fact, stimulation with soluble CD3/CD28 Abs did not elicit any ostensible signaling in tonsillar T-cells, at all, evidencing that tonsillar T-cells exhibit a rather strict requirement for TCR clustering. CD3/CD28 Abs immobilized on latex beads did induce TCR signaling in tonsillar lymphocytes, albeit always weaker than in paired samples from peripheral T-cells. Activation of TCR signaling in the various clinical settings by immobilized CD3/CD28 was roughly of the same magnitude, although T-cells from CT exhibited a trend to weaker responses, e.g. at the level of Erk or Akt activation. Interestingly, this effect of a dampened response in CT was more pronounced in peripheral T-cells as compared to tonsillar T-cells. In conclusion, these experiments evidenced that tonsillar T-cells responded to TCR stimulation, but they exhibited an absolute requirement for TCR/co-receptor clustering in order to elicit a signaling reaction.

### Tonsillar T-cells from CT feature low cell proliferation

**Fig 7:**
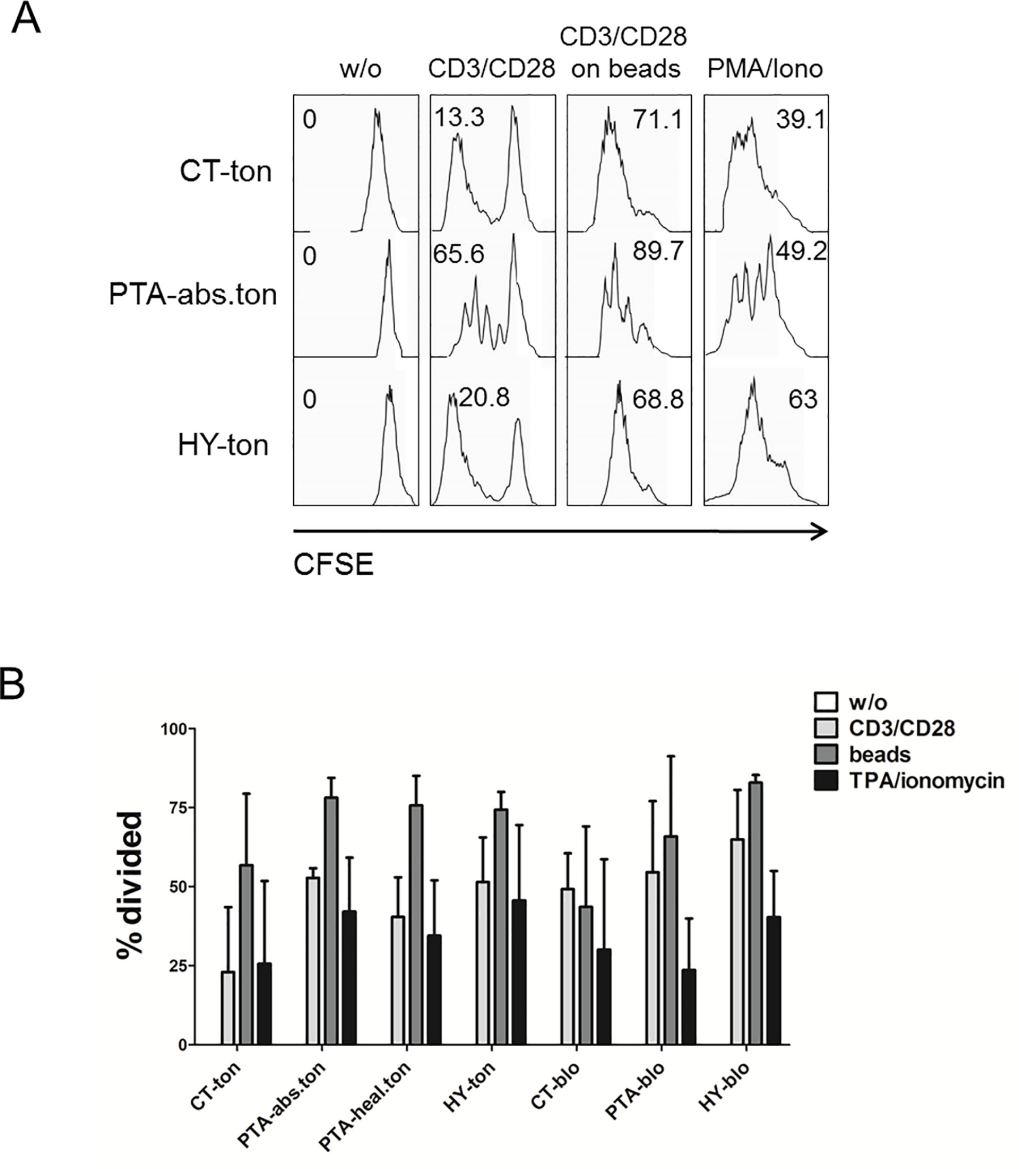
Tonsillar T-cells expand and proliferate in response to TCR activation. **(A)** CD4/CD8 T-cells isolated from tonsils were loaded with the vital dye CFSE and challenged with soluble or immobilized anti-CD3/CD28 Abs to activate the TCR. Cell proliferation of the mixed CD4(+)CD8(+) T-cell preparations was scored as a reduction in CFSE fluorescence as T-cells divide and segregate the dye to daughter cells. Shown here are representative profiles for one sample of each disease group. Insert numbers represent the percentage of all T-cells that underwent at least one cell division. **(B)** Quantification of cell proliferation for all tonsil samples performed with FlowJo software (TreeStar Inc., Ashland, USA). For statistical evaluation, see S1 Table. CT = chronic tonsillitis; PTA = peritonsillar abscess; HY = tonsillar hyperplasia; ton = tonsil; blo = blood; abs = abscess; hea = healthy.

A physiologically appropriate T-cell activation culminates in the clonal expansion of the challenged T-cell clones. We investigated the capacity of tonsillar T-cells to proliferate in response to CD3/CD28 surface clustering/activation by assessing CFSE dilution. The cell permeable dye CFSE is taken up by T-cells and its fluorescence drops by about one half with each round of cell division as the dye segregates into the daughter cells. As illustrated by the representative experiments shown in Fig 7A and the quantification over all samples shown in Fig 7B, proliferation was less pronounced in response to soluble CD3/CD28 Ab stimulation as compared to bead-immobilized Abs, in agreement with the pattern of TCR-dependent signalling (Fig 6A) and with the general notion that TCR clustering by immobilized Abs is a more efficient and physiological T-cell stimulus [31]. Intriguingly, tonsillar T-cells exhibited a proliferative index that was comparable in magnitude to the one of their peripheral T-cell counterparts despite the markedly weaker response to TCR stimulation seen at other levels (e.g. at the level of receptor proximal signalling, Fig 6). Of note, tonsillar T-cells from CT showed the lowest proliferative score of all groups, although this difference did not attain statistical significance. In summary, we conclude that while T-cells from tonsillar infection scenarios are in principle capable of mounting a proliferative response in response to TCR activation, T-cells from CT feature a trend to a reduced response in line with other previous observations that were reminiscent of an exhausted phenotype in this T-cell population.

## Discussion

The role of human palatine tonsils as a secondary immune organ and its contribution to a proper and functional immune response is a highly debated and unsettled issue. Tonsillectomy is a widely applied surgical intervention and the fact that wide-spread deleterious effects are not obvious in tonsillectomized patients, intuitively indicates that tonsils play a minor role, if any, in the immune response to life threatening infections. However, despite the large numbers of tonsillectomies performed daily, there is very few data on the functionality of tonsillar immune cells. Based on current experimental evidence it is hard to judge if tonsillar immune cells are immunologically competent, comparably to their counterparts from the periphery or other lymphoid organs. Previous studies that analyzed T-cells from human palatine tonsils focused on the immune-phenotyping-based characterization of T-cell subclasses or on cytokine release propensity [32–35], but a comprehensive investigation of functional T-cell activation responses to antigenic stimulation has not been performed to date. Moreover, the majority of studies analyzed tonsillar T-cells from children undergoing tonsillectomy as a treatment for tonsil hypertrophy, but there is scarce or no functional data for tonsillar T-cells in other inflammation conditions such as CT.

We have analyzed the response of tonsillar T-cells from chronic tonsillitis and other inflammatory conditions to TCR stimulation. Our initial characterization of tonsillar T-cells subpopulations produced a number of remarkable observations. First, our data confirm previous findings that evidenced a strong overrepresentation of CD4(+) T-cells (about 75% of all T-cells), as compared to the numbers commonly observed in the periphery and other lymphoid organs (range of 1.4 to 2.5). The higher CD4 T-cell ratio was, however, not due to population expansions as a consequence of inflammatory conditions, since it was present in healthy tonsils and non-infectious tissue from HY patients, too. Our findings show that T-cells isolated from human palatine tonsils react to TCR stimulation with a characteristic response that is comparable to that from peripheral T-cells in some aspects, yet features marked differences in other regards. For example, while the general qualitative response to TCR stimulation induced via anti CD3/CD28 Abs as a surrogate TCR stimulus was similar in tonsillar and peripheral T-cells, tonsillar T-cells did exhibit a quantitatively dampened response at the level of proximal TCR signaling. Moreover, we observed that activation of tonsillar T-cells was highly dependent on the clustering of the TCR, since the activation of all investigated signaling pathways became evident only upon addition of immobilized CD3/CD28 Abs, while soluble CD3/CD28 Abs exerted virtually no effect. TCR clustering is known to boost and enable full-blown T-cell activation by antigen presentation in the context of an immunological synapse [36]. Indeed, some TCR-dependent pathways are highly dependent on clustered TCR activation, while other pathways, like e.g. NFAT activation, are much less so [37, 38]. In general, a strict requirement for TCR clustering in the process of T-cell activation is considered a quality trait for T-cells and a safe-guard mechanism that ensures that only APC-dependent antigen presentation will promote a full-blown clonal T-cell activation. Thus, as judged by these observations, tonsillar T-cells would appear to be more “protected” from inappropriate TCR activation. This same phenomenon was observed also at other readout levels, like cell proliferation, where tonsillar but not peripheral T-cells, always exhibited a more pronounced proliferation score if stimulated with immobilized CD3/CD28 Abs compared to soluble Abs. Intriguingly, tonsillar T-cells exhibited robust clonal expansion in response to TCR activation, despite the quantitatively weaker TCR signaling, pointing to lower signalling thresholds for mitogenic signaling in T-cells from tonsils. In sum, we interpret the different sensitivity to TCR clustering between peripheral and tonsillar T-cells and the lower overall signaling intensity in tonsillar T-cells as an indication for qualitatively distinct signaling patterns between both T-cell populations. We are currently designing experiments to dissect the mechanisms in downstream signaling that may account for those differences.

With regard to the differences between the various patient groups, our findings document that T-cells from CT exhibit specific features that distinguish them from PTA or HY, yet alone from peripheral lymphocytes. Overall, T-cells from CT show a tendency to respond weaker to TCR stimulation as assessed at the level of TCR signaling or cell proliferation. This observation is in line with previous studies that reported a state of immune exhaustion or senescence in conditions of chronic inflammation [39]. On the other hand, T-cells from CT show signs of an elevated basal activation status, as reflected by high basal CD69 surface expression, consistent with the notion that tonsillar T-cells are exposed to chronic stimulation in CT. A similar tonic up-regulation of T-cell activation markers has been reported for other settings of chronic or systemic infection such as sepsis [39]. Importantly, it has been reported that chronic exposure to antigen, while initially promoting the accumulation of pathogen-specific effector T-cells, can eventually progress to exhaustion of the affected effector T-cell populations [20–23]. This effect may be reflected, among other features, by the high basal expression of CD69, which has been linked to an exhausted T-cell phenotype in previous studies [40–42]. Such a state of “exhaustion” essentially represents a state of ineptness of the T-cells and may also be the cause for the higher numbers of T-cells expressing high levels of the inhibitory cell surface receptor PD-1 in CT (see Fig 3B, 3C and 3D). Considered together with the recurrent, albeit not significantly weaker responses of T-cells from CT documented herein, the most straightforward interpretation is that T-cells are exposed to an immune-suppressive environment in tonsils from patients with CT.

The potential clinical implications of the data presented here are several-fold. The fact that T-cells form the various pathological tonsillar backgrounds exhibit differences in their functional response to TCR activation is a strong indication that tonsillar lymphocytes sense the various infection scenarios and adapt their effector functions accordingly. This finding is consistent with a role for tonsil palatina in the surveillance and immunological control of local throat infections. In conclusion, our data point out that tonsillar T cells from chronic and acute inflammation settings are functional with respect to their response to TCR challenge with activation and proliferation. These data underscore the notion that tonsils are functional immune entities and suggest that in future tonsillotomy, i.e. only a partial resection of the tonsils, should be discussed and considered more often as an alternative to tonsillectomy in case of CT in adults.

## Supporting information

S1 Table Statistical analysis of tonsil *versus* blood sample results. Comparison of tonsil and blood sample results within individuals stratified by disease and condition with reference to the figures of the main document (exact two-sided p-values of the test).
(DOCX)
