# Supplementary material for: Functional characterization of T-cells from palatine tonsils in patients with chronic tonsillitis
Source: PLoS One. 2017 Sep 6;12(9):e0183214. doi: 10.1371/journal.pone.0183214 (PMC5587326; doi:10.1371/journal.pone.0183214)
Supplement: S1 Table — Comparison of tonsil and blood sample results within individuals stratified by disease and condition with reference to the figures of the main document (exact two-sided p-values of the test). (DOCX) [file pone.0183214.s001.docx]

**Supplementary Table S1:** **Statistical analysis of tonsil *versus* blood sample results.** Comparison of tonsil and blood sample results within individuals stratified by disease and condition with reference to the figures of the main document (exact two-sided p-values of the test).

|  |  |  | CT |  | PTA | |  | HY |
| --- | --- | --- | --- | --- | --- | --- | --- | --- |
|  |  |  | ton vs. blo |  | abs.ton vs. blo | hea.ton vs. blo |  | ton vs. blo |
| Fig 1. (B) total T-cells | % CD4(+) of total T-cells |  | 0.50 |  | 0.03 | -- |  | 0.50 |
|  | CD4(+)/CD8(+) ratio |  | 0.50 |  | 0.03 | -- |  | 0.50 |
| Fig 3. (A) | % regulatory T-cells in all T-cells |  | 0.50 |  | 0.03 | -- |  | 0.50 |
| Fig 7. (B) | w/o |  | -- |  | -- | -- |  | -- |
|  | CD3/CD28 |  | 0.50 |  | 1.00 | 0.50 |  | 0.50 |
|  | coated |  | 0.50 |  | 0.25 | 0.25 |  | 0.50 |
|  | beads |  | 0.50 |  | 0.63 | 0.63 |  | 0.50 |
|  | 3/28/S |  | 1.00 |  | 1.00 | 1.00 |  | 0.50 |
|  | TPA/Ionomycin |  | 0.50 |  | 0.13 | 0.88 |  | 1.00 |
